# Pembrolizumab- and ipilimumab-induced diabetic ketoacidosis and isolated adrenocorticotropic hormone deficiency: a case report

**DOI:** 10.1186/s13256-020-02502-w

**Published:** 2020-09-29

**Authors:** Thachanun Porntharukchareon, Borwonkhun Tontivuthikul, Nattaya Sintawichai, Panudda Srichomkwun

**Affiliations:** 1 Chulabhorn Hospital, HRH Princess Chulabhorn College of Medical Science, Chulabhorn Royal Academy, Bangkok, Thailand; 2 Division of Endocrinology and Metabolism, Department of Medicine, Faculty of Medicine, Chulalongkorn University and the King Chulalongkorn Memorial Hospital, Bangkok, Thailand; 3 Division of Medical Oncology, Department of Medicine, Faculty of Medicine, Chulalongkorn University and the King Chulalongkorn Memorial Hospital, Bangkok, Thailand; 4 Excellent Center in Diabetes, Hormones and Metabolism, King Chulalongkorn Memorial Hospital, Thai Red Cross Society, Bangkok, Thailand

**Keywords:** Immune-related adverse event, Diabetic ketoacidosis, Isolated adrenocorticotropic hormone deficiency, Immunotherapy, Immune checkpoint inhibitors

## Abstract

**Background:**

Several human monoclonal antibodies directed against immune checkpoints, including T lymphocyte antigen 4 and programmed cell death protein 1, have been implemented for cancer treatment in order to promote effector T cell response to tumors. Despite the antitumor activity of these agents, a significant number of patients demonstrated immune-related adverse events that affected the functions of multiple organs, including the endocrine system. We report the first case of immune checkpoint inhibitor–induced simultaneous diabetic ketoacidosis and isolated adrenocorticotropic hormone deficiency following combination treatment with immune checkpoint inhibitors.

**Case presentation:**

A 70-year-old Thai man with no previous history of diabetes mellitus was diagnosed with stage IVB non–small cell lung with pleural and liver metastases. After 14 weeks of combination treatment with pembrolizumab and ipilimumab, he presented with fatigue, nausea, and vomiting. Laboratory investigation revealed random plasma glucose 794 mg/dl, serum ketone 6.3 mmol/L, bicarbonate 13 mmol/L, and high anion gap 24 mmol/L. New-onset diabetes mellitus and diabetic ketoacidosis were diagnosed. Insulin therapy was initiated a favorable outcome within 10 hours. Despite improvement of hyperglycemia, the patient had persistent nausea and hyponatremia. Further investigation revealed cortisol 0.8 μg/dl and adrenocorticotropic hormone 21.7 pg/ml. His other pituitary hormone levels were normal, except for mild elevation of gonadotropin hormone. Magnetic resonance imaging of the pituitary showed a normal pituitary gland. Isolated adrenocorticotropic hormone deficiency was diagnosed, and corticosteroid replacement therapy was administered, resulting in an improvement of his symptoms.

**Conclusion:**

Our patient developed new-onset diabetes mellitus, diabetic ketoacidosis, and isolated adrenocorticotropic hormone deficiency during cancer treatment with pembrolizumab and ipilimumab. The present case highlights the need for physicians to be aware that immune-related adverse events can occur in multiple organs at the same time.

## Background

The modern era of cancer treatment is constantly evolving, with new breakthroughs and discoveries. Immune checkpoint inhibitors (ICIs) are a new and effective class of cancer immunotherapy. Several human monoclonal antibodies directed against immune checkpoints, including cytotoxic T-lymphocyte-associated protein 4 (CTLA-4), program death protein 1 (PD-1), and programmed death-ligand 1 (PD-L1), have been implemented for cancer treatment in order to promote an effector T cell response to tumors. Despite the antitumor activity of these agents, a significant number of patients demonstrated autoimmunity leading to immune-related adverse events (IRAEs). IRAEs can potentially affect functions of multiple organs, including the endocrine system. Hypophysitis, thyroid dysfunction, insulin-deficient diabetes mellitus (DM), and primary adrenal insufficiency have been reported as IRAEs due to ICI therapy [1]. Insulin-deficient DM and adrenal insufficiency are infrequent ICI-related endocrinopathies but can result in life-threatening diabetic ketoacidosis (DKA) or adrenal crisis, respectively, without early diagnosis and appropriate management. In this report, we present a case of ICI-induced simultaneous DKA and isolated adrenocorticotropic hormone deficiency (IAD) following combination treatment with pembrolizumab and ipilimumab in a patient with advanced non–small cell lung cancer (NSCLC).

## Case presentation

A 70-year-old Thai man was diagnosed with stage IVB NSCLC with pleural and liver metastases. The patient’s medical history was unremarkable until December 2018, when he had right pruritic chest pain and nonproductive cough. The patient had smoked cigarettes (20 packs per year) and did not have a drinking habit. The patient had good performance status, and he was not taking any medications. He was found to have high levels of PD-L1 expression (tumor proportion score ≥ 50%) but had a negative result for epidermal growth factor receptor mutations and anaplastic lymphoma kinase rearrangement. He was treated with a combination of pembrolizumab 200 mg every 3 weeks and ipilimumab 1 mg/kg every 6 weeks. A combination of PD-1 and CTLA-4 inhibitors was used as a first-line treatment in this patient because the available evidence suggested that combination therapy may produce a higher tumor response rate than PD-1 inhibitors alone in metastatic NSCLC PD-L1 positive subgroups [2].

After 14 weeks of treatment, the fifth cycle of pembrolizumab and the third cycle of ipilimumab, he presented with complaints of fatigue, vigorous nausea, and vomiting without abdominal pain. His physical examination revealed his body temperature was 37.2 °C, blood pressure was 100/60 mmHg, pulse rate was 116 beats per minute, and respiratory rates was 20 breaths per minute. The examination of his abdomen, neurological system, and other systems was unremarkable.

The patient’s random plasma glucose level was 794 mg/dl, and his serum ketone level was 6.3 mmol/L. His arterial blood gas analysis showed a pH of 7.17. He was admitted to the hospital, and the results of further laboratory investigations are shown in Table 1. Hyperglycemia, high serum ketone, low bicarbonate at 13 mmol/L, and high anion gap at 24 mmol/L were compatible with the DKA criteria. A treatment protocol for DKA with aggressive intravenous hydration and continuous intravenous insulin was initiated with a favorable outcome within 10 hours.

**Table 1:** Laboratory data of the patient

Investigation	1 month before admission	This admission	6 months after admission	Reference range
Plasma glucose, mg/dl	87	**794**	**210**	70–99
HbA1C, %	N/A	**6.5**	**7.0**	4.0–5.6
Beta-hydroxybutyrate, mmol/L	N/A	**6.3**	N/A	0–0.6
Sodium, mmol/L	135	**119**	140	136–145
Potassium, mmol/L	3.3	**6.5**	3.4	3.4–4.5
Chloride, mmol/L	97	82	99	95–105
Bicarbonate, mmol/L	28	**13**	26	22–29
Anion gap, mmol/L	10	**24**	15	8–16
Blood urea nitrogen, mg/dl	5	**39**	N/A	7–20
Creatinine, mg/dl	0.75	**1.8**	0.82	0.7–1.2
Amylase, U/L	N/A	60	N/A	20–100
Lipase, U/L	N/A	53	N/A	10–60
Anti-GAD, IU/ml	N/A	3.09	N/A	< 17
Anti-IA2, IU/ml	N/A	< 7.5	N/A	< 7.5
C-peptide	N/A	**< 0.1**	N/A	0.9–7.1
8:00 a.m. cortisol, ng/ml	N/A	**0.6**	N/A	3–18
ACTH, pg/ml	N/A	21.7	N/A	0–46
FT4, ng/dl	N/A	1.22	1.19	0.8–1.8
TSH, μIU/ml	N/A	2.94	2.560	0.3–4.1
FSH, IU/L	N/A	**17.7**	13.9	1–8.4
LH, IU/L	N/A	**19.4**	8.1	1–10.5
Testosterone, nmol/L	N/A	24.46	14.89	5.9–24.7
IGF-1, ng/ml	N/A	N/A	66.4	24.6–269
Prolactin, ng/ml	N/A	15.9	6.7	2–25

*Abbreviations: ACTH* Adrenocorticotropic hormone, *FSH* Follicle-stimulating hormone, *FT4* Free thyroxine, *GAD* Glutamic acid decarboxylase, *IA2* Islet antigen 2, *IGF-1* Insulin-like growth factor 1, *LH* Luteinizing hormone, *N/A* Not available, *TSH* Thyroid-stimulating hormone Boldface define abnormal value

Boldface define abnormal value

The diagnosis of ICI-related DM was suspected due to the abrupt onset of DM. Blood analysis revealed an undetectable C-peptide level and a negative result of glutamic acid decarboxylase autoantibodies (anti-GAD) and anti–tyrosine phosphatase-like islet antigen 2.

**Fig. 1:**
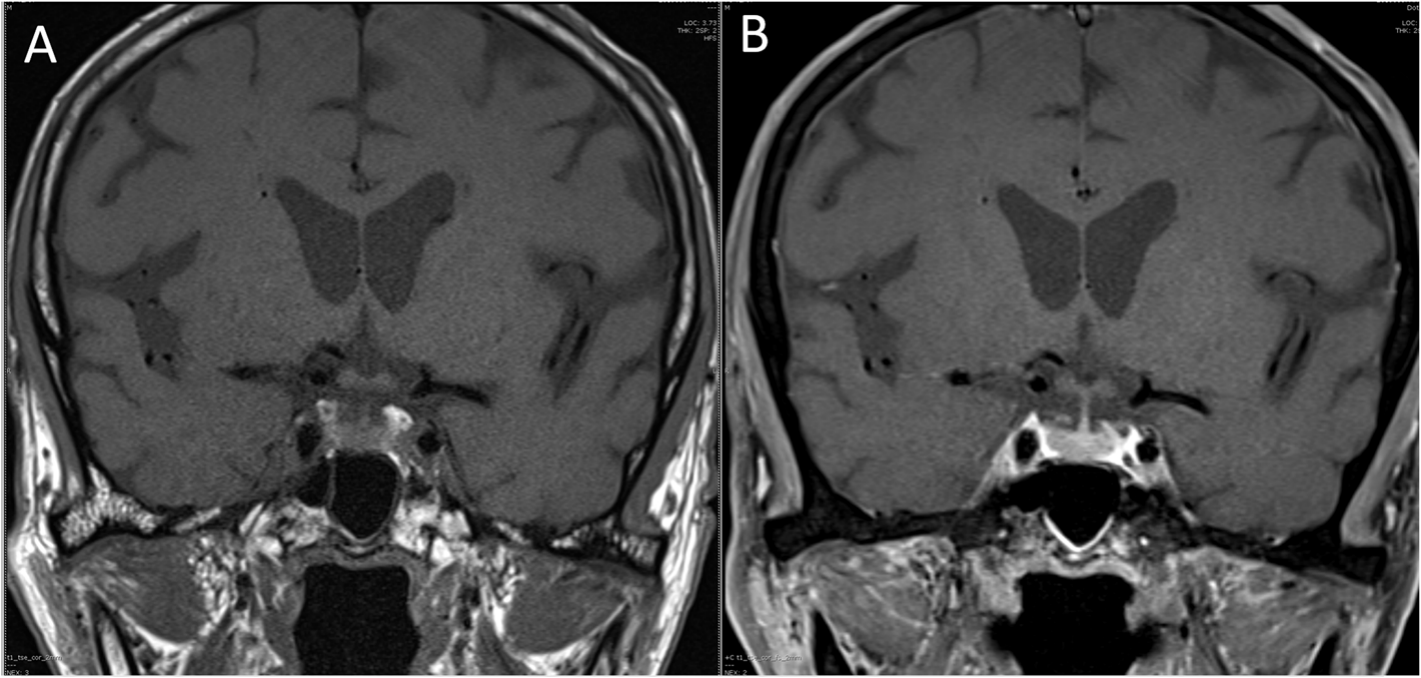
Magnetic resonance imaging (MRI) of the pituitary gland, coronal plane. **a** T1-weighted MRI showing no abnormalities in the pituitary gland, hypophyseal stalk, and hypothalamus. **b** Gadolinium-enhanced T1-weighted MRI showing a symmetric, round-shaped pituitary gland with homogeneous enhancement of the hypophyseal stalk

Despite his hyperglycemia improving, he still had persistent nausea and hyponatremia (serum sodium 126 mmol/L). Further investigations revealed a very low morning cortisol level (0.8 μg/dl) and normal adrenocorticotropic hormone (ACTH) level (21.7 pg/ml; normal range 0–46). His other pituitary hormone levels were normal, except for mild elevation of follicle-stimulating hormone/luteinizing hormone (Table 1). He was diagnosed with IAD and immediately received intravenous hydrocortisone. Magnetic resonance imaging (MRI) of the pituitary showed a normal pituitary gland (Fig. 1).

Twenty-four hours after starting corticosteroid replacement, his symptoms and hyponatremia resolved. He was then switched from hydrocortisone to prednisolone. He was discharged on the 12th day of admission with prednisolone 7.5 mg/day and premixed insulin 26 U/day. Both his pembrolizumab and ipilimumab were discontinued due to IRAEs.

Six months following hospital admission, he was seen in regular follow-up in the endocrinology department. His blood glucose levels were borderline controlled with premixed insulin 58 U/day (Table 1). He remained on prednisone 7.5 mg/day, and he felt extremely fatigued if he missed a dose of prednisolone. He had stable disease even after ICI discontinuation and no further treatment.

## Discussion

ICIs are approved for many types of cancer. Pembrolizumab is a PD-1 inhibitor approved for melanoma, NSCLC, small cell lung cancer, renal cell carcinoma, and head and neck squamous cell carcinoma. Ipilimumab is a CTLA-4 inhibitor approved for melanoma, renal cell carcinoma, microsatellite instability–high, or mismatch repair deficiency colorectal cancer [1]. Combination therapy with CTLA-4 inhibitors and PD-1/PD-L1 inhibitors have proved beneficial in advanced NSCLC, but they are limited by their serious side effects [2]. ICI-related endocrinopathies resulting from combination therapy are hypothyroidism (5.6–22.5%), hyperthyroidism (6.6–16.1%), thyroiditis (3.8–4.6%), hypophysitis (6.2–16.4%), primary adrenal insufficiency (1.2–36.8%), and DM (2%) [3].

Our patient presented with DKA after the fifth cycle of pembrolizumab and the third cycle of ipilimumab with fulminant onset, undetectable C-peptide, and negative autoimmune DM antibodies. ICI-related DM is a rare but potentially life-threatening IRAE. Most ICI-related DM cases have been due to PD-1/PD-L1 inhibitors rather than CTLA-4 inhibitors [1]. The incidence rates of DM in patients treated with only PD-1/PD-L1 inhibitors and a combination of PD-1/PD-L1 inhibitors plus CTLA-4 inhibitors are estimated at 1% and 2%, respectively [3, 4].

Several important features characterize ICI-related DM: (1) abrupt onset of hyperglycemia, (2) rapid progression of endogenous insulin deficiency, and (3) high risk of DKA if not detected and treated promptly with insulin therapy [5]. De Filette *et al.* reported that DKA is the first clinical presentation in 71% of patients with ICI-related DM [5]. Autoimmunity is the main hypothesized pathogenesis of ICI-related DM; however, the conclusions remain unclear because half of the patients with ICI-related DM have a negative finding for autoimmune DM antibodies [1]. Time of presentation is inconsistent with a median time of onset of 3.1 cycles (range, 1–17) for patients with positive anti-GAD and 5.9 cycles (range, 1–16) for patients with negative anti-GAD findings, which is similar to the case of our patient [5]. Although the management of ICI-related DM is long-term insulin treatment, Hansen *et al.* reported one patient with ICI-related DM who was able to discontinue insulin and recovered from the C-peptide level after ICI discontinuation [6]. The limitation of our patient’s case is the lack of information about genetic factors or human leukocyte antigen genotypes of the patient that may predispose to endocrine IRAEs [5, 7].

Several patients who developed ICI-related DM developed IRAEs prior to, concurrent with, or subsequent to the development of ICI-related DM, such as hypophysitis and thyroid dysfunction [1]. Our patient also has another endocrinopathy. He had a low cortisol level without elevated levels of ACTH and normal function of other pituitary axes, supporting a diagnosis of IAD.

ICI-related IAD is a rare endocrinopathy. Percik *et al.* reported that the prevalence of ICI-related IAD was 0.87% in their retrospective cohort study of all patients with cancer treated with ICI [8]. The risk for developing IAD was four times higher among women than among men and seven times higher in patients treated with combined PD-1/PD-L1 inhibitors plus ipilimumab than in patients treated with only PD-1/PD-L1 inhibitors (3.2% vs. 0.4%, respectively) [8]. In contrast to most autoimmune hypophysitis cases, IAD onset ranges from approximately 4 to 8 months, with a median period of 6 months, which appears to be longer than that for hypophysitis (approximately 2 months) [7]. Although the mechanism of the pathophysiology of ICI-related IAD has not been elucidated, the development of antipituitary autoantibodies and the direct effects of CTLA-4 inhibitors on the pituitary are considered [9]. In contrast to conventional hypophysitis, the MRI findings of ICI-related IAD mostly showed no enlargement of the pituitary gland, similar to the case of our patient [7]. The main treatment is long-term glucocorticoid replacement in a physiologic dose [8].

According to the latest version of the National Cancer Institute’s Common Terminology Criteria for Adverse Events [10], our patient had a grade 4 adverse event for DKA and a grade 3 adverse event for ICI-related IAD. The treatment with ICI should be discontinued until DM is controlled and adequate hormone replacement therapy is administered [11]. ICI was discontinued in our patient due to IRAEs and poor performance status.

## Conclusion

To the best of our knowledge, this is the first case report of ICI-induced DM/DKA and IAD following the combination treatment of PD-1 and CTLA-4 inhibitors. The present case highlights the need for physicians to be aware that IRAEs can occur in multiple organs at the same time.

## Data Availability

All data generated during this study are included in this published article.

## Abbreviations

ACTH: Adrenocorticotropic hormone
CTLA-4: Cytotoxic T-lymphocyte-associated protein 4
DKA: Diabetic ketoacidosis
DM: Diabetes mellitus
FSH: Follicle-stimulating hormone
FT4: Free thyroxine
GAD: Glutamic acid decarboxylase
IA2: Islet antigen 2
IAD: Isolated adrenocorticotropic hormone deficiency
ICI: Immune checkpoint inhibitor
IGF-1: Insulin-like growth factor 1
IRAE: Immune-related adverse event
LH: Luteinizing hormone
MRI: Magnetic resonance imaging
N/A: Not available
NSCLC: Non–small cell lung cancer
PD-1: Programmed death 1
PD-L1: Programmed death-ligand 1
TSH: Thyroid-stimulating hormone
